# Childhood Trauma and Social Skills in Residential Care Youth: the Roles of Coping Strategies and Self-efficacy

**DOI:** 10.1007/s40653-026-00875-6

**Published:** 2026-04-20

**Authors:** Mălina-Ionela Corlătianu, Cornelia Măirean

**Affiliations:** Alexandru Ioan Cuza University, Iasi, Romania

**Keywords:** Childhood traumatic experiences, Perceived social skills, Coping strategies, Self-efficacy, Residential care

## Abstract

This study explored how childhood traumatic experiences, such as abuse and neglect, are related to perceived poor social skills. It also examined how coping strategies and self-efficacy may moderate that relationship. The sample consisted of 214 participants (55.6% girls; Mage = 15.27) living in residential care in Romania. The length of time spent in the residential care center varies between one month and 16 years, with a mean of 4.81 years (*SD* = 3.80). Participants completed measures assessing traumatic experiences, perceived social skills, coping strategies, and self-efficacy. Path analysis showed that traumatic experiences and maladaptive coping strategies positively predicted, while adaptive coping strategies negatively predicted perceived poor social skills. This relationship was moderated only by self-efficacy. When self‑efficacy was high, participants with high levels of traumatic experiences also reported higher levels of perceived poor social skills. Among participants with minimal trauma exposure, low self-efficacy was still associated with poorer social skills. Lacking confidence in one’s ability to manage social situations can be a barrier to healthy interpersonal functioning, even without a history of significant trauma. These findings highlight the importance of strengthening adaptive coping and building self-efficacy in all children. Both are associated with stronger socio-emotional development and show different patterns of association with adversity.

Children in residential care are highly vulnerable due to prior trauma, increased risks of developmental issues, and unmet mental health needs. Research shows that between 19% and 40% of these youths suffer from post‑traumatic stress disorder (PTSD), and over 90% have experienced trauma (Lawrence-Sidebottom et al., 2024; Lewis et al., 2019). Many children in residential care have experienced repeated placement disruptions and often enter the system following severe neglect, abuse, or family breakdown (Harder et al., 2012). Across Europe, the number of children living in residential settings remains substantial, with 337,287 currently placed in such care (United Nations Children’s Fund [UNICEF], 2024).

Children living in residential care face a higher likelihood of experiencing social-emotional challenges, such as difficulties in social interactions and ineffective coping mechanisms (Fernando et al., 2024). These issues are frequently intensified by instability in caregiving relationships, which can impede emotional healing and growth (Göbbels-Koch & Gupta, 2025; Weiler et al., 2022). Stable, empathetic relationships with residential staff can act as protective factors, supporting social skills, emotional regulation, and resilience (Gallardo‑Masa et al., 2024). However, youths in residential care remain vulnerable to disruptions in mentoring relationships, which can worsen trauma symptoms and reinforce unhealthy relational patterns (Harder et al., 2012; Weiler et al., 2022).

Emerging research also highlights that coping and self‑efficacy function differently for youth in residential care compared to children raised in stable family environments. Youth in residential care often report lower levels of self‑efficacy, partly due to limited opportunities for autonomy and repeated experiences of disempowerment in early caregiving relationships (Harden, 2004; Leve et al., 2012). Similarly, studies show that youth in residential care tend to rely more on maladaptive coping strategies, such as avoidance, emotional suppression, or self‑blame, especially when their early environments have been unpredictable or emotionally unsafe (Compas et al., 2017; Kim & Cicchetti, 2010). At the same time, when residential care settings provide stable, supportive relationships, young people demonstrate greater use of adaptive coping and show improvements in emotional regulation and social functioning (Greeson et al., 2011).

These findings underscore why coping and self‑efficacy are particularly relevant factors for understanding social outcomes among youth in residential care. This research addresses an important gap in existing literature by focusing on the less-explored group of youth in residential foster care and emphasizing the protective role of coping strategies and self-efficacy. Given the limited availability of family-like alternatives and the institutional nature of care in Romania, it is essential to examine how trauma experiences are linked to social functioning in these settings. A deeper understanding can help shape more effective mental health interventions and inform national child welfare policies.

## Childhood Traumatic Experiences and Perceived Social Skills

Meaningful social interactions depend on a set of learned skills that allow individuals to respond to others in flexible, thoughtful, and emotionally aware ways (Herrera Torres & Bravo Antonio, 2012). These social skills include the ability to express emotions, needs, opinions, and personal boundaries, while adapting to the demands of different situations and relationships (Caballo et al., 2015). Early childhood is a key period for developing these, which emerge through daily routines and peer interactions (Del Prette & Del Prette, 2013). These foundational skills support relationship‑building, emotional regulation, and coping, and are especially important for children in challenging environments (Beenen et al., 2023; Carriedo et al., 2024). Matson and colleagues (2010) identify six dimensions of social skills, ranging from assertiveness and communication problems to impulsive or withdrawn behavior and difficulties in relationships.

Childhood trauma can take many forms, including physical, emotional, and sexual abuse, as well as neglect (Slep et al., 2015; Suniega et al., 2022), and often occurs within relationships marked by trust or power, causing significant harm to a child’s health, development, and overall well-being (World Health Organization, 2020). The prevalence of such experiences varies widely across continents: emotional abuse affects between 6% and 61% of children, physical abuse between 22% and 60%, sexual abuse between 6% and 27%, and neglect between 17% and 65% (Moody et al., 2018). Trauma can shape how children feel, behave, and relate to others, especially when painful experiences occur repeatedly or over long periods, keeping the body in a prolonged state of stress that makes daily life more difficult (Teicher & Samson, 2016).

Furthermore, trauma has been associated with heightened risk of aggressive behavior (Zielinski & Bradshaw, 2006), peer victimization, and peer rejection, particularly during the school years (Kawabata et al., 2024; Xiao et al., 2021; Zheng et al., 2025). Moreover, adults who went through traumatic events early in life tend to report less satisfaction in their romantic partnerships, face higher rates of separation and divorce, conflicts, violence, and may struggle with parenting (Labella et al., 2018; Savage et al., 2019). These relational vulnerabilities are often accompanied by a more fragile sense of self, difficulties in trusting others, and challenges in feeling welcomed in new environments (Hepp et al., 2018).

Studies show that trauma can slow the development of social skills, including the ability to calm down, solve problems, and communicate clearly when something feels wrong (Copeland et al., 2018; Sarafim-Silva & Bernabé, 2021; Tyler et al., 2021). Young people with strong trauma symptoms often struggle in social situations, finding it difficult to connect with others or to feel understood (Roberts et al., 2013). Children who have been maltreated are often perceived by both peers and adults as less socially competent, and they tend to struggle with initiating and sustaining friendships (Bolger & Patterson, 2001; Do & Widom, 2025). In addition, neglected children are more likely to withdraw socially and face lower levels of peer acceptance (Hildyard & Wolfe, 2002; Pfaltz, 2022). Studies focusing on children in residential care indicate consistent social‑emotional difficulties: caregivers describe reduced social understanding, challenges in recognizing and responding to others’ emotions, and less coherent empathic reactions (Luke & Banerjee, 2012; Rubin et al., 2012). They also tend to engage in less complex and cooperative interactions, struggling to initiate and sustain social exchanges (Smyke et al., 2007).

## The Role of Coping Strategies

Monat and Lazarus (1991, p. 5) describe coping as how individuals respond to situations they perceive as harmful, threatening, or challenging, especially when those demands feel overwhelming or beyond their personal resources. Coping strategies are often grouped into two broad categories: problem‑focused coping, which involves efforts to address or change the source of stress, and emotion‑focused coping, which refers to managing the emotional response to the stressor (Compas et al., 1993). Coping also involves a cognitive *versus* behavioral dimension: some strategies focus on how we think, others on what we do (Wang et al., 2024). Cognitive coping strategies, also known as cognitive emotion regulation, refer to how people mentally analyze and moderate emotional responses to stressful experiences (Thompson, 1991; Zagaria et al., 2023). These strategies are generally viewed as stable patterns of responding to adversity, though they can change across development and through therapeutic or supportive interventions.

Garnefski et al. (2002) distinguish nine types of cognitive coping strategies: (1) Self-blame involves holding oneself responsible for negative events, often fueling guilt and shame (Anderson et al., 1994; Robison et al., 2025); (2) Acceptance involves acknowledging what has happened and mentally letting go of the need to change it, even when the outcome is painful (Carver et al., 1989; Segal et al., 2025); (3) Rumination means getting mentally stuck in the emotions and thoughts tied to a negative event, often replaying them without resolution (Mancone et al., 2025; Nolen-Hoeksema et al., 1994); (4) Positive refocusing involves shifting attention away from the stressful event and instead thinking about pleasant or uplifting things to ease emotional distress (Endler & Parker, 1990; Stanisławski et al., 2025); (5) Refocusing on planning involves mentally shifting toward the steps we can take to manage or respond to the stressful event, emphasizing action and preparation (Carver et al., 1989; Zhang, 2024); (6) Positive reappraisal involves finding personal growth or meaning in a difficult experience (Carver et al., 1989; Meyers et al., 2025); (7) Putting into perspective means mentally placing the event in a broader context, often by comparing it to more serious situations, which can help reduce its emotional weight (Allan & Gilbert, 1995; Chaaya et al., 2025); (8) Catastrophizing means mentally exaggerating the impact of a negative event, often imagining worst-case scenarios and feeling overwhelmed by fear (Sullivan et al., 1995); (9) Blaming others involves mentally assigning responsibility for a negative event to someone else, often as a way to make sense of what happened or to protect oneself from feelings of guilt (Robison et al., 2025; Tennen & Affleck, 1990).

These strategies are often grouped into adaptive (acceptance, positive refocusing, refocusing on planning, positive reappraisal, putting into perspective) and maladaptive (self‑blame, rumination, catastrophizing, blaming others) categories, based on their associations with psychological adjustment (Carver, 1997; Compas et al., 2017).

Children learn to manage emotions and stress by observing the adults around them, but when early relationships are marked by trauma, this learning process can be disrupted. As a result, many children grow up struggling to solve problems effectively or regulate their emotions (Edossa et al., 2018; Morris et al., 2014). This means that children who’ve lived through trauma often need extra support not just to understand and manage their emotions, but also to learn healthy ways of coping and to practice socially appropriate behavior in everyday situations (Edossa et al., 2018).

Adaptive strategies support long‑term well‑being, whereas maladaptive ones may offer short‑term relief but undermine emotional health over time (Rodrigues et al., 2023). For adolescent girls especially, the way they cope can shape how trauma shows up, whether through emotional dysregulation, avoidance, or struggles in relationships. When coping is adaptive, it can soften trauma‑related symptoms; when it is not, distress often deepens (Elzy et al., 2013; Jenkins et al., 2021; Zhang et al., 2025). Within this framework, avoidant coping may offer short‑term relief by reducing immediate emotional intensity, whereas approach‑oriented strategies (such as emotional processing or direct engagement with traumatic memories) do not moderate the association between trauma exposure and symptom severity in adolescent girls with histories of complex trauma. These findings highlight the nuanced nature of coping and the need for personalized interventions. Tailoring support to individual coping styles may be key to promoting long-term healing (Elzy et al., 2013; Zhang et al., 2025).

Strengthening coping can enhance emotional resilience and improve the ability to manage future challenges. Research on youth in residential care shows that many have experienced multiple traumatic events and continue to struggle with emotional distress and PTSD symptoms (Eth, 2000; Salazar et al., 2013). These difficulties often impair emotion regulation, stress management, and everyday social functioning. Young people who rely on more adaptive coping strategies tend to show greater emotional stability and healthier relationships (Greeson et al., 2011; Salazar et al., 2013). Supporting the development of adaptive coping is therefore essential in residential care, as it can foster resilience and better prepare youth for future challenges.

## The Role of Self-efficacy

Self‑efficacy refers to people’s beliefs about their ability to organize and execute the actions required to achieve specific goals (Bandura, 1977). It shapes how individuals approach challenges, stay engaged, and respond when things become difficult (Schunk, 1995). These beliefs determine problem‑solving, motivation, emotional management, and decision‑making, making self‑efficacy a central factor in how individuals adapt, grow, and pursue meaningful goals (Benight & Bandura, 2004; Yang & Delgado, 2025). Social self‑efficacy supports connection and relationship‑building, academic self‑efficacy guides how students approach learning, and emotional self‑efficacy helps individuals manage stress and difficult emotions. Together, these domains shape how people navigate daily demands (García‑Álvarez et al., 2021; Muris, 2001). Grounded in Albert Bandura’s foundational work, self-efficacy theory highlights that mastery experiences, modeling, social persuasion, and emotional states work together to build the confidence we need to act. When self‑efficacy is strong, individuals show greater flexibility, growth, and a stronger sense of success across life domains (Bandura, 1977; Phan & Ngu, 2016).

Research on children’s self-efficacy has largely focused on typical developmental pathways. When children feel confident in their abilities, especially in social and academic settings, they are more likely to form strong peer relationships and succeed in school (Bandura et al., 1996; Remondi et al., 2023). Their social self-perception also shapes how they behave with others, directly influencing the quality of their friendships and interactions (Rubin et al., 2012). Children who have experienced maltreatment often carry invisible wounds that affect how they see themselves and how they relate to others (Chen et al., 2024). When these children enter school, they may struggle with self-regulation, have difficulty focusing or planning, and find it hard to build trusting relationships (Bartlett & Smith, 2019; Morin, 2020). A strong sense of social efficacy can serve as a protective factor as it fosters confidence, supports clear self-expression, and helps build meaningful connections with others (Li et al., 2023).

When someone experiences traumatic stress, self-efficacy often becomes a guiding force in their recovery (Benight & Bandura, 2004; Murphy et al., 2025). When individuals trust their ability to cope, they are more likely to engage in the healing process and regain a sense of control over their lives (Cieslak et al., 2008). In the early school years, younger children who have experienced abuse often report feeling more competent and more accepted by peers than they are (Flynn et al., 2023; Kim & Cicchetti, 2003). This overestimation may reflect a form of defensive processing, a way to protect their sense of self and maintain a sense of control. As children grow older, however, this protective lens tends to fade. By grades 4 to 6, abused children often describe themselves as less competent and less accepted than their non-abused peers, revealing a shift toward more negative self-perceptions (Flynn et al., 2023; Kim & Cicchetti, 2003).

Evidence shows that self-efficacy plays a key moderating role in the connection between childhood trauma and later social development. When children believe in their ability to interact and build relationships, they are more likely to adapt well and show resilience, even after difficult early experiences (Benight & Bandura, 2004; von Wendorff et al., 2025). In this way, self-efficacy helps protect children from the lasting effects of trauma. Individuals with higher self-efficacy tend to approach social interactions with more confidence, especially during moments of tension or disagreement. This sense of assurance allows them to manage conflict in a more constructive and emotionally balanced way (Treat et al., 2020). Over time, it supports the development of stronger and more stable relationships (Kim & Cicchetti, 2003).

The Intergenerational Trauma Treatment Model (ITTM; Scott & Copping, 2008) places self-efficacy at the center of healing across generations. This theoretical framework suggests that change across generations begins when children and caregivers rebuild a sense of safety, agency, and connection, processes that rely strongly on strengthening self‑efficacy. When children who have experienced trauma begin to trust their ability to cope and connect with others, they often show progress in emotional regulation and social competence. Strengthening self‑efficacy in both children and caregivers can enhance relationships, emotional adjustment, and long‑term well‑being (Copping, 2018).

For children in residential care, who often enter school with lower self‑efficacy due to earlier traumatic experiences, strengthening their sense of competence can play a crucial protective role, support emotional adjustment, and help them build more stable and trusting relationships with peers and caregivers (Greeson et al., 2011; Salazar et al., 2013).

## The Current Study

Although abuse and neglect within residential foster care has been well documented as having negative relationships with mental health (Lueger‑Schuster et al., 2018), there is still limited empirical research examining the relationship between traumatic experiences and children’s social functioning in these settings. This gap in understanding makes it more difficult to design strategies and interventions that genuinely support young people who have lived through trauma. To address this gap, the present study examines the association between childhood traumatic experiences and poorer social skills among children and adolescents living in residential foster care. By exploring this link, we aim to inform the development of evidence‑based practices that respond to the distinct socio‑emotional needs of this population, one of the most vulnerable and least represented groups in trauma research. In addition, we examine whether coping strategies and self‑efficacy moderate the association between childhood traumatic experiences and social skills.

Based on the literature reviewed above (Compas et al., 2017; Kim & Cicchetti, 2010; Luszczynska et al., 2009), we anticipated that: (1) Childhood traumatic experiences positively predict perceived poor social skills; (2) Coping strategies (adaptative strategies, maladaptive strategies) moderate the relationships between childhood traumatic experiences and perceived poor social skills. Specifically, for those who adopt maladaptive coping strategies there is stronger negative relationship between childhood traumatic experiences and perceived poor social skills, while for the participants who report high level of adaptive coping strategies, the relation between childhood traumatic experiences and perceived poor social skills is weaker; (3) Self-efficacy moderates the relationships between childhood traumatic experiences and perceived poor social skills. Specifically, those with low levels of self-efficacy show a stronger positive relationship between childhood traumatic experiences and perceived poor social skills.

## Method

### Participants and Procedure

The initial sample consisted of 223 participants (54.1% girls; *M* age = 15.20). In line with the study’s focus on adolescents, we restricted the analyses to participants aged 12–18 years. As a result, nine participants below this age range (2 aged 9, 3 aged 10, 6 aged 11) and six participants above it (aged 19–21) were excluded. The final analytic sample included 214 participants (55.6% girls), drawn from eight residential foster care centers belonging to the General Directorate of Social Assistance and Child Protection, Romania. In the Romanian context, residential foster care centers host children who have experienced various forms of adversity, including neglect, abandonment, and abuse, and who often lack consistent family-based support. Although youth in residential foster care face increased vulnerability, systematic research into their socio-emotional development remains limited. In the current sample, 53.3% of participants came from families living in rural areas. Their ages ranged from 12 to 18 years, with a mean age of 15.27 (SD = 1.67), reflecting a wide developmental span. Educational attainment varied across the group: 65% had completed middle school, 4.7% had finished elementary school, and 30.4% had graduated from high school. These figures point to diverse academic paths within this population. The length of time spent in the residential care center varies between one month and 16 years, with a mean of 4.81 years (*SD* = 3.80).

The Research Ethics Committee has approved the proposed research study (No. 1654/24.10.2023). The first author contacted each foster care center via email, subsequently followed by in-person meetings with the principals to present the objectives of the research and to inform the psychologists about the particularities of the scales applied and the process of data collection. The residential care centers involved in the study were located in the northeastern and southern regions of Romania. Out of the 16 foster care centers contacted, 11 provided a positive response and agreed to participate in the study. In each center, it was decided that the administration of the scales would be conducted by a person familiar to the children, namely the center’s psychologist. During group meetings held in each center, the psychologists were trained regarding the data collection procedure. Alongside the institutional approval granted by the principals of the participating centers, each participant was invited to sign an informed consent form, confirming their voluntary involvement in the study.

The informed consent process was conducted using age-appropriate language, and participants were encouraged to ask questions before deciding whether to take part. They were clearly informed that all collected data would remain confidential and be used solely for research purposes. Participants were also assured of their right to withdraw from the study at any time, without any consequences or obligations. No compensation was offered to participants for their participation in the study. The administration of the scales was conducted in small groups of participants and required approximately 40 min to complete. The participating foster care centers accommodated children and adolescents without diagnosed intellectual disabilities, ensuring that all participants had the ability to read and complete the questionnaire items independently. Additionally, the psychologists offered psychological counseling to the participants at their request, if their involvement in the study resulted in psychological distress. The scales were completed in the following order: Childhood Trauma Questionnaire, Matson Evaluation of Social Skills with Youngsters Scale, The Cognitive Emotion Regulation Questionnaire, The Self-Efficacy Questionnaire for Children.

### Measures

Matson Evaluation of Social Skills with Youngsters Scale, Self-Report version (MESSY; Matson, 1988) is a measure of social skills that includes 64 items rated on a 5‑point Likert scale ranging from 1 (not at all) to 5 (very much). The scores from the Appropriate Social Skills subscale are reversed. This adjusted score is then added to the raw score from the Inappropriate Social Skills subscale. A high total score indicates poor social skills. Existing research shows that the scale has strong psychometric qualities in terms of internal consistency, as well as both convergent and divergent validity (e.g., Matson et al., 2010). Previous studies report Cronbach’s alpha values typically ranging from 0.80 to 0.90 for the total score (Matson et al., 1985, 2010). In our sample, the measure has an adequate internal consistency for total score (Cronbach’s α = 0.92).

Childhood Trauma Questionnaire (CTQ; Bernstein & Fink, 1998) is a tool designed to assess the presence and nature of childhood traumatic experiences. The instrument is a 28-item retrospective self-report questionnaire that assesses experiences occurring during childhood and adolescence. Items are rated on a 5‑point Likert scale ranging from 1 (Never True) to 5 (Very Often True). Higher scores indicate higher levels of traumatic experiences. The CTQ has demonstrated excellent test-retest reliability in previous studies, with values ranging from 0.79 to 0.81 (Bernstein & Fink, 1998; Bernstein et al., 1994). The scale has also demonstrated convergent validity, with clinician-rated interviews of childhood abuse and therapist ratings of abuse (Bernstein & Fink, 1998; Bernstein et al., 1997). In our study, the measure showed adequate internal consistency for total score (Cronbach’s α = 0.89).

The Cognitive Emotion Regulation Questionnaire, Romanian version (CERQ; Garnefski et al., 2002) was used to evaluate the cognitive dimension of emotion regulation. The CERQ is a 36-item self-report questionnaire that utilizes a 5-point Likert scale ranging from 1 (almost never) to 5 (almost always). It assesses nine distinct cognitive emotion regulation strategies grouped into adaptive (acceptance, positive refocusing, refocusing on planning, positive reappraisal, and putting into perspective) and maladaptive (self-blame, rumination, catastrophizing, and blaming others) strategies (Jermann et al., 2009; Willem et al., 2019). Previous studies report good internal consistency for the CERQ subscales, with Cronbach’s alpha values typically ranging from 0.75 to 0.87 (Garnefski et al., 2002; Jermann et al., 2009). In the current study, we used two scores, one for adaptative strategies, and one for maladaptive strategies. This scoring approach is consistent with prior psychometric work indicating that CERQ subscales tend to cluster into two higher‑order dimensions reflecting broader adaptive and maladaptive regulatory tendencies, with this line of work also reporting adequate reliability for both dimensions (Domínguez‑Sánchez et al., 2013; Martin & Dahlen, 2005). Using composite scores reduces model complexity and multicollinearity among the nine subscales while capturing theoretically meaningful patterns of cognitive emotion regulation in adolescents. In the present study, the internal reliability indices for adaptive and maladaptive strategies were very good (0.87 and 0.84, respectively).

The Self-Efficacy Questionnaire for Children (SEQ-C; Muris, 2001) is composed of 24 items, which are hypothesized to represent three domains of self-efficacy, namely: (1) social self-efficacy, pertaining to the individual’s perceived ability to initiate and maintain peer relationships and to act assertively in social contexts; (2) academic self-efficacy, concerning the perceived ability to regulate their learning, master academic content, and meet educational demands; and (3) emotional self-efficacy, relating to the perceived ability to manage and cope with negative emotions effectively. Each item is rated on a 5‑point Likert scale ranging from 1 (not at all) to 5 (very well). A total self-efficacy score was calculated by summing across all items. Using the total score is consistent with prior work, indicating that global self‑efficacy reflects a broad sense of personal competence that is strongly linked to adolescents’ socio‑emotional functioning. The total score also demonstrates stronger psychometric stability than the individual domains and avoids inflating model complexity through multiple, highly correlated subscales (Muris, 2001; Schwarzer & Jerusalem, 1995). Previous research shows that the SEQ‑C total score demonstrates good to excellent internal consistency, with Cronbach’s alpha values typically ranging from 0.85 to 0.90 across adolescent samples (Caprara et al., 2010; Muris, 2002; Muris et al., 2016). In our study, the measure has an adequate internal consistency for total score (Cronbach’s α = 0.91).

### Overview of Statistical Analysis

**Fig. 1:**
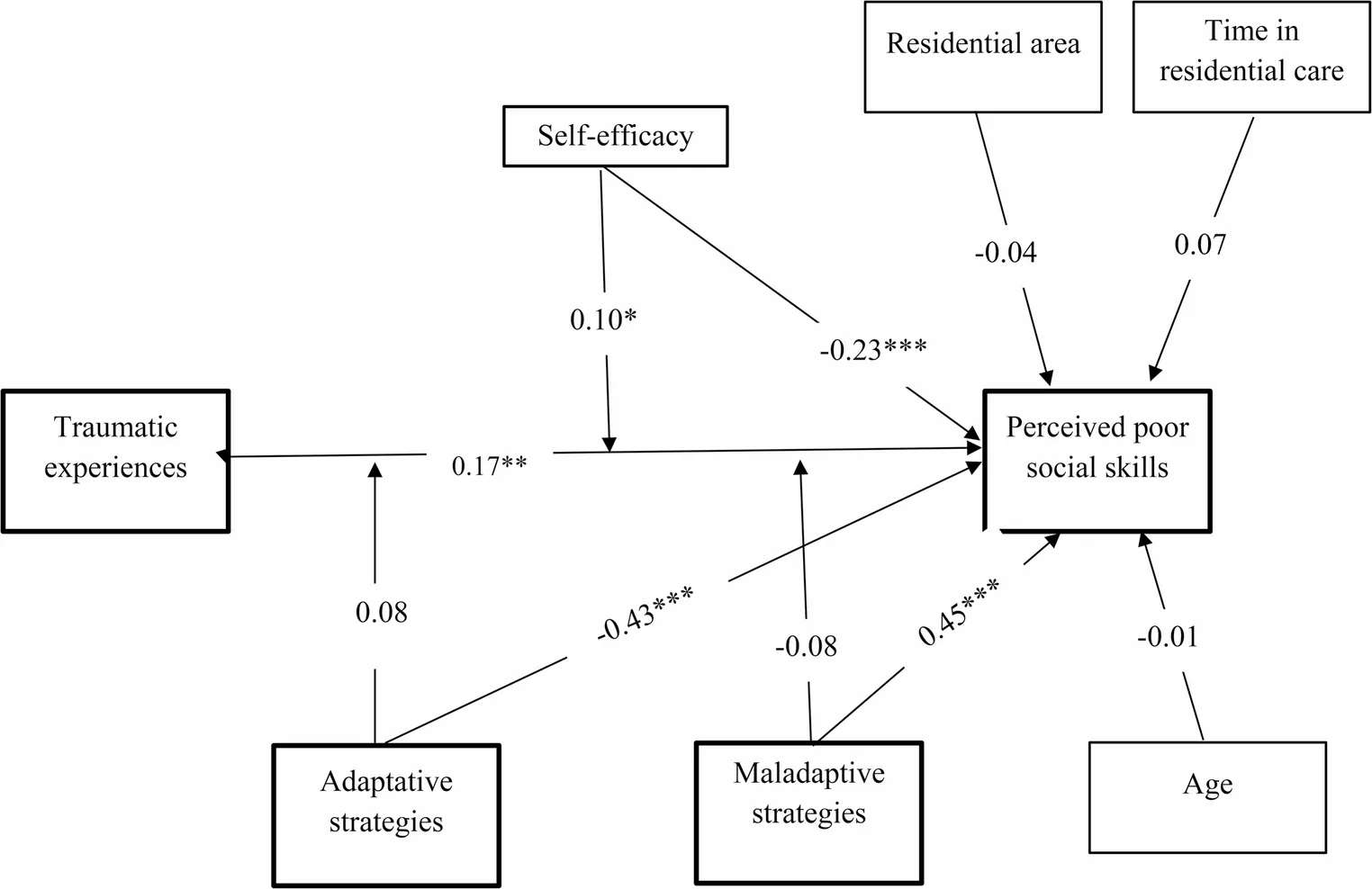
Structural equation model and path analysis of the predictors of perceived poor social skills. (*N* = 214). Standardized path coefficients reported. *p ≤.05; **p ≤.01; ***p <.001

First, we computed means, standard deviations, and checked the normality distribution of data as preliminary statistics, using the SPSS 29 software. We also used independent sample t-tests and One-Way Analysis of Variance to analyze the differences between participants based on gender and education level, concerning poor social skills. Then, we calculated descriptive statistics and zero-correlations between the main variables. After that, we used a structural equation model (SEM) framework in AMOS Graphics 29 to simultaneously test the relation between childhood traumatic experiences with perceived poor social skills, as well as the moderating role of coping strategies (adaptative strategies, maladaptive strategies) and self-efficacy. Interaction terms were included as observed variables in the SEM. The residential area, age, and the length of time spent in the residential care was dummy-coded and entered as covariates into the model predicting the dependent variable. Estimates were obtained via maximum likelihood, and overall model fit was evaluated using normative fit index (NFI), goodness of fit (GFI), the comparative fit index (CFI), and the root mean square residual (RMSEA). To graphically display the interaction terms, we used Dawson’s (2014) method (see Fig. 1). All continuous predictors were mean-centered prior to creating interaction terms. Interaction terms were computed by multiplying the centered predictor and moderator variables. Significant interactions were probed using simple slope analyses, with slopes examined at − 1 SD (low), the mean (medium), and + 1 SD (high) of the moderator, providing both graphical and statistical interpretation of the moderation. No missing data were present in the variables included in the analyses.

## Results

### Preliminary Analysis and Associations between Variables

The results of the descriptive statistics analysis indicated that the data were normally distributed (see Table 1), with skewness values ranging from − 0.11 to 0.55 and kurtosis values ranging from − 0.76 to 0.03, supporting the suitability of the variables for parametric analyses.

**Table 1:** Pearson correlation for the study variables and descriptive statistics analysis

Variables	(1)	(2)	(3)	(4)	(5)	(6)	(7)	(8)
1. Traumatic experiences	1							
2. Adaptive strategies	0.07	1						
3. Maladaptive strategies	0.40***	0.43***	1					
4. Self-efficacy	− 0.19**	0.37***	0.02	1				
5. Perceived poor social skills	0.33***	− 0.33***	0.29***	− 0.42***	1			
6. Time in residential care	− 0.13	0.02	− 0.21**	0.05	− 0.03	1		
7. Age	0.05	0.07	− 0.02	0.005	− 0.02	0.22***	1	
8. Residential area	0.07	0.10	0.03	0.01	− 0.07	0.45	− 0.03	1
9. Mean	60.5	65.13	42.06	91.12	138.79	4.81	15.27	1.57
10.SD	21.31	34.41	10.35	21.31	32.92	3.80	1.67	0.82
11.Min	27	28	19	24	76	1	12	1
12.Max	121	96	75	134	225	16	18	2

*p ≤.05; **p ≤.01; ***p <.001. *N* = 214

There are statistical differences between participants from urban and rural areas in perceived poor social skills (*t* (212) = 2.002; *p* =.04), with those from urban areas reporting higher levels of perceived poor social skills (*M =* 143.57). There are no statistical differences between male and female participants in perceived poor social skills (*t* (212) = 0.30; *p* =.76). In terms of educational attainment, there are also no statistical differences in perceived poor social skills (*F* (2,211) = 0.39; *p* =.67).

Pearson correlation analysis (see Table 1) showed that perceived poor social skills were positively related to traumatic experiences (*r* =.33; *p* <.001) and maladaptive strategies (*r* =.29; *p* <.001). Further, perceived poor social skills were negatively related to adaptive strategies (*r* = −.33; *p* <.001) and self-efficacy (*r* = −.42; *p* <.001). There was no significant association between perceived poor social skills and time in residential care (*r* = −.03; *p* =.66). Regarding age, we observed a non-significant correlation with perceived poor social skills (*r* =.02; *p* =.80).

### The Path Analysis

The fit for our overall model is good (Fig. 1): χ2 (44) = 92.295, *p* <.001; NFI = 0.79; CFI = 0.87; RMSEA = 0.07 (CI: 0.05, 0.09). The model explained 47.7% of the variance in perceived poor social skills.

Traumatic experiences positively predicted perceived poor social skills (*β* = 0.17, *p* =.003). When analyzing the role of adaptative strategies, the results showed that adaptative strategies were negatively related to perceived poor social skills (*β* = − 0.43, *p* <.001), but did not moderate the relation between traumatic experiences and perceived poor social skills (*β* = − 0.08, *p* =.16). Furthermore, the findings indicate that maladaptive strategies were positively related to perceived poor social skills (*β* = 0.45, *p* <.001) but did not moderate the relation between traumatic experiences and perceived poor social skills (*β* = − 0.08, *p* =.15). Additionally, the results suggest that self-efficacy was negatively related to perceived poor social skills (*β* = − 0.23, *p* <.001), and it moderated the relation between traumatic experiences and perceived poor social skills (*β* = 0.10, *p* =.04). Moreover, the residential area did not predict perceived poor social skills (*β* =- 0.04, *p* =.40), nor did age (*β* =- 0.01, *p* =.83) or time in residential care (*β* = 0.07; *p* =.14).

**Fig. 2:**
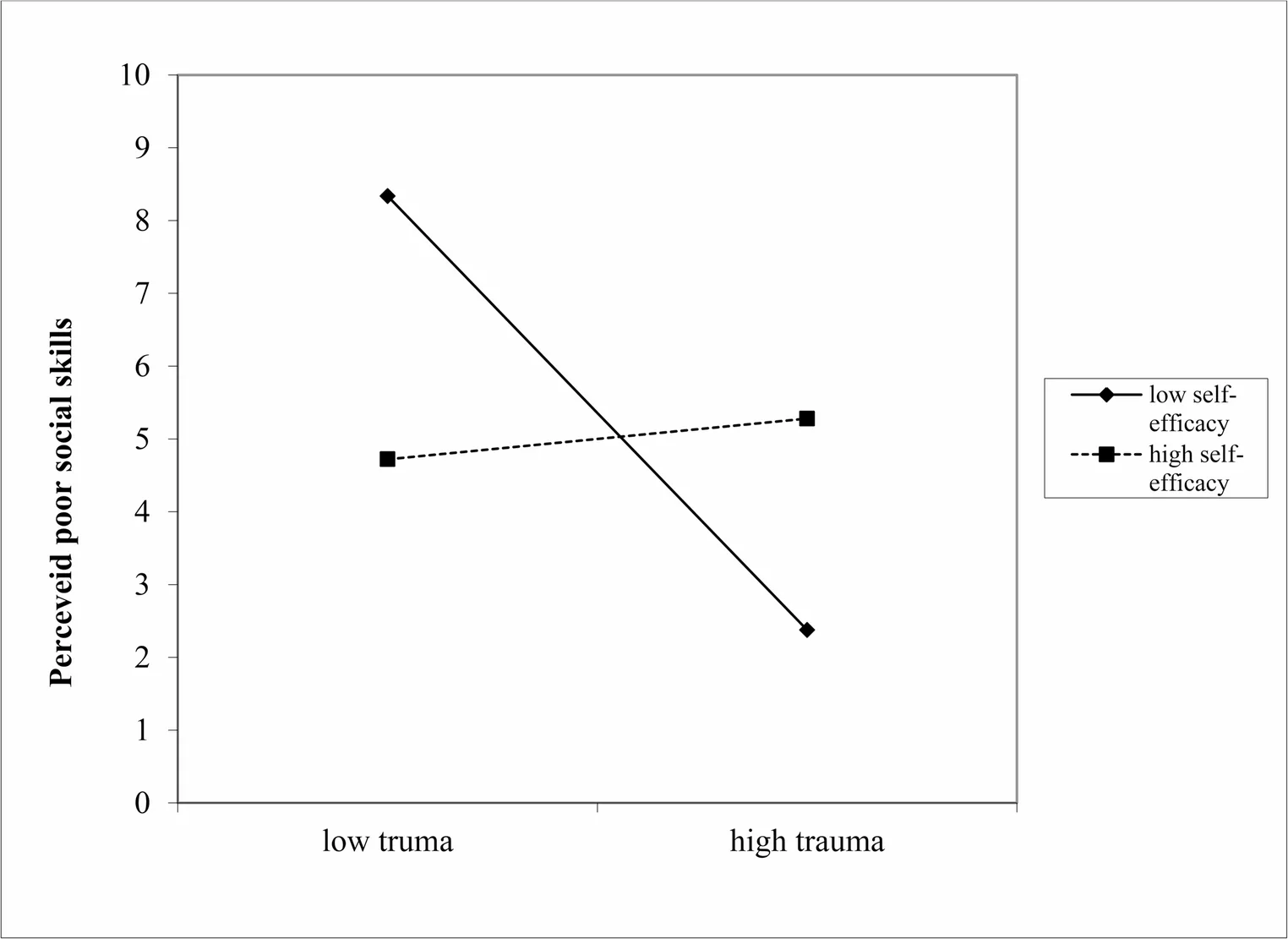
The moderating role of self-efficacy in the relation between traumatic childhood experiences and perceived poor social skills

The results indicated that the association between traumatic experiences and perceived poor social skills differed depending on the level of self‑efficacy. The simple slope analysis indicated that the interaction was significant only at high levels of self-efficacy (*t =* 2.94, *p =*.004), whereas the slope at low levels of self-efficacy was not significant (*t =* − 1.56, *p =*.118; see Fig. 2). When self‑efficacy was high, participants with high levels of traumatic experiences also reported higher levels of perceived poor social skills.

## Discussion

The present study aimed to investigate the associations between childhood traumatic experiences and perceived poor social skills, as well as the moderating roles of adaptive strategies, maladaptive strategies, and self-efficacy.

Our findings showed that childhood trauma is positively related to perceived poor social skills. This aligns with previous research showing that early adversity can disrupt both emotional development and social adjustment (Haslam & Taylor, 2022; Zielinski & Bradshaw, 2006). Children exposed to trauma may misinterpret social cues or find it hard to trust others, which can lead to tense or ineffective communication (Haslam & Taylor, 2022). Evidence from residential care settings further supports this pattern: children raised in institutional environments, where early deprivation and relational instability are common, consistently show difficulties in social communication, peer relationships, and trust-building (Smyke et al., 2007). Early trauma heightens stress sensitivity and encourages coping patterns such as withdrawal or aggression (Zielinski & Bradshaw, 2006). Taken together, these studies show how trauma shapes not only a child’s inner emotional world but also the way they connect with those around them.

Further, adaptive strategies are negatively related to perceived poor social skills, indicating that youths who rely more on acceptance, positive refocusing, refocusing on planning, positive reappraisal, and putting into perspective tend to experience lower levels of disrupted social abilities. This pattern is consistent with research showing that adaptive coping and effortful control support emotional regulation and prosocial behavior across diverse developmental contexts (Compas et al., 2017; Eisenberg et al., 2001). However, adaptive coping did not moderate the association between traumatic experiences and perceived poor social skills. This aligns with evidence suggesting that coping strategies often operate as broad resilience resources rather than as factors that alter the role of specific stressors (Compas et al., 2017). One explanation is that the benefits of adaptive coping depend on the presence of stable relational and environmental support. In contexts where such forms of support are limited, such as residential care, children may continue to display social communication difficulties despite using seemingly adaptive strategies, reflecting the cumulative effects of early deprivation and inconsistent caregiving (Smyke et al., 2007).

In contrast, maladaptive coping strategies were positively associated with poor social skills, consistent with findings that avoidance, rumination, and emotional suppression contribute to social withdrawal, conflict, and reduced peer competence (Braun‑Lewensohn, 2015; Eisenberg et al., 2001). Yet, similar to adaptive coping, these strategies did not moderate the link between traumatic experiences and social functioning, supporting the view that maladaptive coping exacerbates interpersonal difficulties without altering the underlying association between trauma and social outcomes (Compas et al., 2017). These findings highlight the importance of interventions that reduce reliance on maladaptive coping and strengthen regulatory and interpersonal skills to support socio‑emotional development.

Self‑efficacy plays an important role in this study, being negatively related to poor social skills and moderating the relationship between trauma and perceived poor social skills. However, the pattern diverges from our initial hypothesis: although high self‑efficacy is typically linked to better socio‑emotional functioning, youths with high trauma exposure and higher self‑efficacy reported poorer social skills. This suggests that the protective effects of self‑efficacy may not operate uniformly in contexts of severe or chronic trauma. One explanation is that general self‑efficacy, as measured in this study, reflects a broad sense of competence that may not translate into the specific regulatory and interpersonal skills required after trauma. Meta‑analytic evidence indicates that coping‑specific self‑efficacy (i.e., beliefs about one’s ability to manage stressors and regulate emotions) is more strongly associated with resilience in trauma‑related contexts than general self‑efficacy (Gallagher et al., 2020).

Thus, trauma‑exposed youths may feel generally capable while still struggling with trust, emotional regulation, or interpreting social cues, difficulties shaped by trauma‑related beliefs, attachment patterns, and stress responses (Calhoun et al., 2022; Morison & Benight, 2022). Similar patterns are documented among children raised in residential care, who often display persistent socio‑emotional difficulties despite appearing competent in certain areas, reflecting the long‑term effects of early deprivation and relational instability (Humphreys et al., 2015; McLaughlin et al., 2017). In contrast, when self‑efficacy was low, differences in perceived social skills across levels of trauma exposure were not statistically significant, suggesting that variations in trauma exposure do not translate into meaningful differences in perceived social functioning for youths with low self‑efficacy.

These findings have meaningful implications for how trauma is addressed in both practice and policy. By showing the moderating role of self‑efficacy, the study highlights the need for interventions that move beyond symptom relief and instead build concrete psychosocial resources that help children regulate emotions, strengthen relationships, and develop the confidence to face challenges. In residential foster care settings, where emotional and relational vulnerabilities are more pronounced, integrating such skill‑building programs can make a lasting difference.

The results also underscore the importance of early prevention and a strength‑based, individualized clinical approach, one that views each child not only through the lens of their difficulties but also through their capacity to heal, grow, and thrive. Taken together, the findings point to the need for interventions that go beyond strengthening general self‑efficacy and instead focus on calibrating self‑beliefs and developing coping‑specific competencies, particularly for youths exposed to high levels of trauma, where self‑efficacy may operate in more complex and less protective ways. Future studies could examine whether coping‑specific self‑efficacy offers a more precise and clinically relevant indicator of resilience in trauma‑exposed youth, and whether interventions targeting this form of efficacy yield stronger improvements in social outcomes.

This study has several limitations. First, because the data is cross-sectional, it does not allow for firm conclusions about cause and effect. Participants also reported past traumatic experiences retrospectively, which means their responses may have been shaped by memory distortions or recall bias. Another limitation is the lack of information about the timing and type of trauma, factors that strongly shape how individuals respond and adapt. Future research should examine these aspects using longitudinal designs to clarify how trauma influences socio‑emotional development over time.

A further limitation concerns the way cognitive emotion regulation was operationalized. Although the use of adaptive and maladaptive CERQ composite scores is supported by prior research (e.g., Domínguez‑Sánchez et al., 2013; Garnefski et al., 2002), collapsing the nine subscales into two broad categories may obscure meaningful differences between specific strategies. Evidence suggests that individual regulatory strategies can function differently depending on developmental stage and context, which the composite scores cannot fully capture (Aldao et al., 2010; Compas et al., 2017). For instance, strategies such as acceptance or rumination may have adaptive effects in some situations but maladaptive consequences in others, depending on the demands of the environment and the adolescent’s developmental capacities. Future research should therefore examine individual CERQ strategies to clarify their context-dependent roles.

A related limitation concerns the measurement of self‑efficacy. Although the total score provides a reliable and theoretically coherent indicator of general perceived competence, it may not fully capture domain‑specific aspects that are particularly relevant for social functioning. Prior research suggests that social self‑efficacy can play a distinct role in shaping interpersonal behavior and peer relationships (Muris, 2001; Smith & Betz, 2000). Because the present study relied solely on the global score, it cannot determine whether domain‑specific self‑efficacy would have shown different or stronger associations with social skills. Future studies should therefore incorporate more differentiated measures of self‑efficacy to clarify these distinctions. Another limitation relates to the wide age range of the sample. Additionally, other psychological factors, such as perceived stress, resilience, and relationships quality, were not included, despite their relevance for socio‑emotional outcomes, especially in foster care populations. Considering these factors could offer a more layered and nuanced understanding of how young people navigate adversity and develop social competence.

In conclusion, this study offers fresh insight into how childhood trauma is related to social skills in adolescents living in residential care. By showing how coping strategies and self-efficacy are related to poor social skills, the findings underscore the powerful role of psychological resilience. Beyond confirming what earlier studies have shown, these findings highlight specific areas that can be actively supported through prevention and therapy. Understanding how these factors work is essential for designing interventions that promote lasting socio-emotional well-being. Future research should build on these insights by exploring other protective factors, including stress tolerance and broader traits linked to resilience. This would help create more responsive and comprehensive support systems for vulnerable youth th and help them grow in ways that feel sustainable and meaningful.

## Data Availability

The data that support the findings of this study are available from the corresponding author upon reasonable request.
